# Unexpected Variation in Neuroanatomy among Diverse Nematode Species

**DOI:** 10.3389/fnana.2015.00162

**Published:** 2016-01-05

**Authors:** Ziduan Han, Stephanie Boas, Nathan E. Schroeder

**Affiliations:** ^1^Department of Crop Sciences, University of Illinois at Urbana-Champaign, UrbanaIL, USA; ^2^Neuroscience Program, University of Illinois at Urbana-Champaign, UrbanaIL, USA

**Keywords:** invertebrate, amphid, phasmid, *Pratylenchus*, Meloidogyne, *Heterodera*, Heterorhabditis, heterochrony

## Abstract

Nematodes are considered excellent models for understanding fundamental aspects of neuron function. However, nematodes are less frequently used as models for examining the evolution of nervous systems. While the habitats and behaviors of nematodes are diverse, the neuroanatomy of nematodes is often considered highly conserved. A small number of nematode species greatly influences our understanding of nematode neurobiology. The free-living species *Caenorhabditis elegans* and, to a lesser extent, the mammalian gastrointestinal parasite *Ascaris suum* are, historically, the primary sources of knowledge regarding nematode neurobiology. Despite differences in size and habitat, *C. elegans* and *A. suum* share a surprisingly similar neuroanatomy. Here, we examined species across several clades in the phylum Nematoda and show that there is a surprising degree of neuroanatomical variation both within and among nematode clades when compared to *C. elegans* and *Ascaris*. We found variation in the numbers of neurons in the ventral nerve cord and dye-filling pattern of sensory neurons. For example, we found that *Pristionchus pacificus*, a bacterial feeding species used for comparative developmental research had 20% fewer ventral cord neurons compared to *C. elegans*. *Steinernema carpocapsae*, an insect-parasitic nematode capable of jumping behavior, had 40% more ventral cord neurons than *C. elegans*. Interestingly, the non-jumping congeneric nematode, *S. glaseri* showed an identical number of ventral cord neurons as *S. carpocapsae*. There was also variability in the timing of neurodevelopment of the ventral cord with two of five species that hatch as second-stage juveniles showing delayed neurodevelopment. We also found unexpected variation in the dye-filling of sensory neurons among examined species. Again, sensory neuron dye-filling pattern did not strictly correlate with phylogeny. Our results demonstrate that variation in nematode neuroanatomy is more prevalent than previously assumed and recommend this diverse phylum for future “evo-devo-neuro” studies.

## Introduction

For the past 200 years, nematodes received significant attention from neurobiologists due to their relatively simple anatomy (reviewed in Chitwood and Chitwood, 1938). The nervous system of the nematode *Caenorhabditis elegans* consists of only 302 neurons in the adult hermaphrodite and remains the only nervous system to be completely reconstructed (White et al., 1986). In addition to *C.* *elegans*, the neuroanatomy of several parasitic and free-living (non-parasitic) species has been examined using both light and transmission electron microscopy (TEM).

**FIGURE 1:**
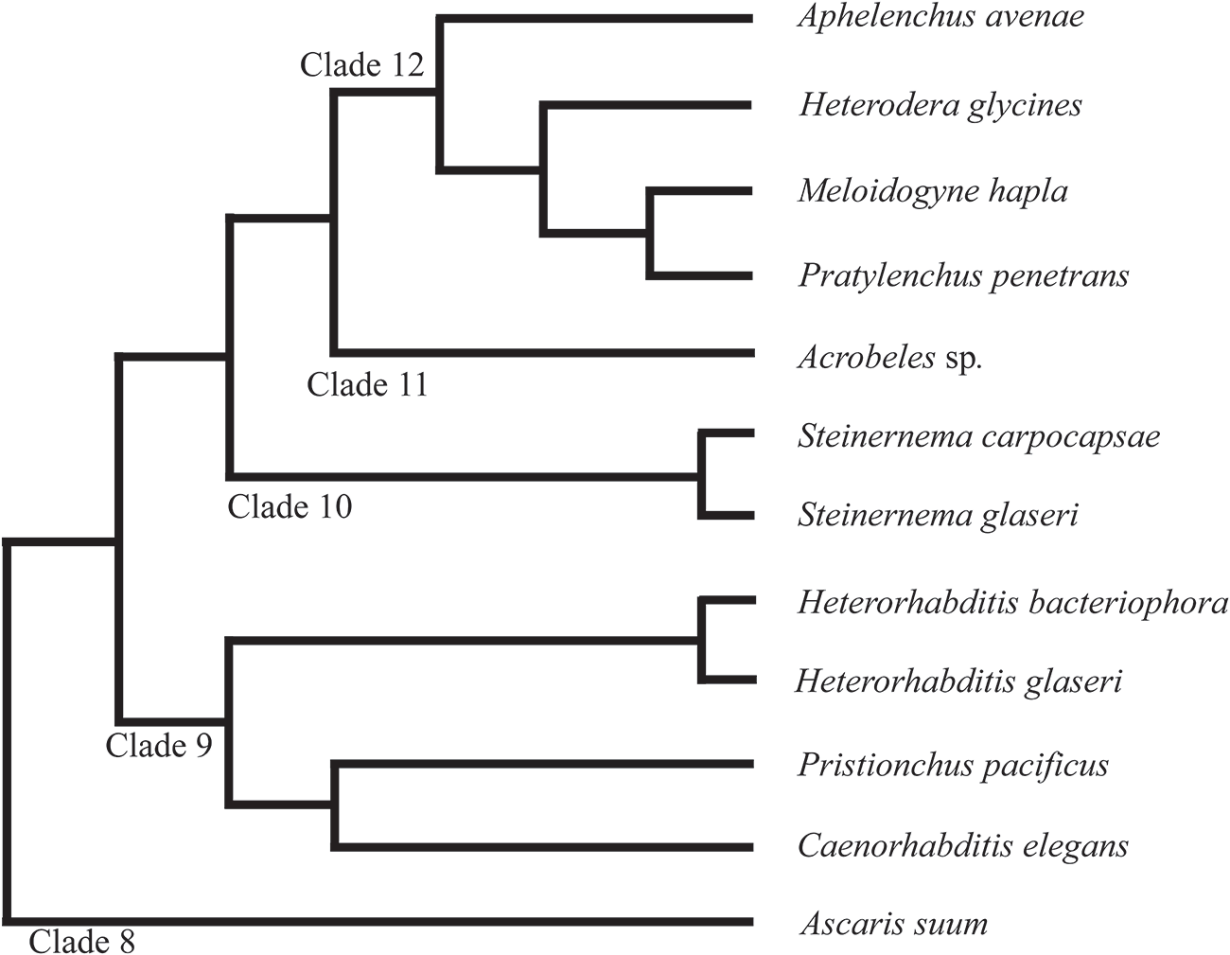
**Phylogeny of nematode species discussed in this study.** The phylum Nematoda is currently divided into 12 clades as discussed in van Megen et al. (2009). Branch lengths do not represent distance.

The phylum Nematoda is currently divided into 12 Clades (**Figure 1**) (Holterman et al., 2006; van Megen et al., 2009). Significant divergence in neuroanatomy exists between nematodes in basal clades (class Enoplea; formerly Adenophorea) and those in higher clades (class Chromadorea; formerly Secernentea) (Sulston and Horvitz, 1977; Gans and Burr, 1994; Malakhov, 1994). However, within the higher clades (clades 8–12), which include *C. elegans* and other intensely studied species, the neuroanatomy is often considered highly conserved (Angstadt et al., 1989; Martin et al., 2002; Burr and Robinson, 2004; Kimber and Fleming, 2005; Hallem and Sternberg, 2008; Srinivasan et al., 2008).

A classic example supporting a high degree of conservation of neuroanatomy among nematodes is the similarity in structure between the ventral nerve cords (VNC) of *C. elegans* (Clade 9) and the gastrointestinal parasitic nematode *Ascaris suum* (Clade 8). The VNC consists of a series of motor neurons that innervate body-wall muscles and regulate movement (White et al., 1976). While *Ascaris* adults are hundreds of times larger than *C. elegans* and inhabit an extremely different environment, the number of ventral cord neurons is remarkably similar. In *C. elegans*, 57 neurons in the VNC innervate 95 body-wall muscles (Sulston, 1976; White et al., 1976). In *A. suum*, 55 neurons in the VNC innervate approximately 50,000 muscle cells (Stretton, 1976; Stretton et al., 1978).

While the VNC anatomy of other nematode species has received little attention, several studies have examined the anatomy of anterior sensory neurons and pharyngeal neurons (Ward et al., 1975; Ashton and Schad, 1996; Endo, 1998; Li et al., 2001; Bumbarger et al., 2009; Ragsdale et al., 2009). In *C. elegans*, there is one pair of amphid sensilla each containing 12 sensory neurons. The anterior nervous system of *C. elegans* also contains six inner labial, six outer labial and four cephalic sensilla each with an invariant number of neurons (Ward et al., 1975). While the pattern of sensilla and underlying neurons is typically conserved among examined species, variations in the number, position and ultrastructure of the anterior nervous system are well documented (Endo, 1998; Ashton et al., 1999; Bumbarger et al., 2009; Ragsdale et al., 2009). Similarly, recent data demonstrated extensive differences in neuronal connectivity between the pharynxes of *C. elegans* and *Pristionchus pacificus* (Bumbarger et al., 2013), a Clade 9 nematode frequently used for evo-devo studies. These anatomical differences may underlie functional differences in feeding behavior between the two species (Chiang et al., 2006).

To elucidate the evolution of nematode nervous systems, we utilized Differential Interference Contrast (DIC) and fluorescence microscopy to examine the neuroanatomy of the VNC and sensory neurons in nematodes from clades 9 to 12 (Holterman et al., 2006; van Megen et al., 2009). We found unexpected variation in the number of putative neurons in the VNC as well as the dye-filling pattern of chemosensory neurons among several species of parasitic and free-living nematodes. The variability was found both within and among nematodes clades suggesting a dynamic evolution of nematode neuroanatomy. Furthermore, we found variation in the developmental timing of the VNC among nematode species. Our results suggest that nematodes represent a valuable resource for understanding the evolution of nervous systems.

## Materials and Methods

### Nematode Cultures

*Meloidogyne hapla* was isolated by the senior author from infected tomato plants and identified using morphological characters. *Pratylenchus penetrans* was isolated by Dr. Terry Niblack (formerly University of Illinois). Both *M. hapla* and *P. penetrans* were cultured on monoxenic excised corn and tomato root cultures, respectively (Lauritis et al., 1983). Seeds for monoxenic cultures were surface sterilized and germinated on water agar. After germination, roots were excised and transferred to Gamborg’s agar (Gamborg et al., 1968; Rebois and Huettel, 1986). *Heterodera glycines* was received from the plant clinic at University of Illinois and maintained in a sandy loam soil on the soybean variety ‘Lee’ in the greenhouse. *Aphelenchus avenae* was originally isolated by the senior author from soil surrounding garlic plants and identified using morphological characters. *A. avenae* was cultured on ¼ strength Potato Dextrose Agar with the fungus *Botrytis cinerea* as previously described (De Soyza, 1973). The entomopathogenic species (*Steinernema* sp. and *Heterorhabditis* sp.) were received from Dr. Albrecht Koppenhöfer at Rutgers University and reared on living greater wax moth larvae *Galleria mellonella* (Carolina Biological Supply Company, Burlington, NC, USA; Kaya and Stock, 1997). Infective juveniles (IJs) of the four species were collected using White traps (White, 1927) and stored in cell culture flasks with water before DAPI staining. Two methods were used to collect non-IJ and adult stages of entomopathogenic nematodes. For *S. carpocapsae*, IJs were induced to recover and complete development on lipid agar plates as previously described (Wouts, 1981). For other EPNs, non-IJs were collected by dissecting open *Galleria mellonella* approximately 9 days after inoculation. This allows for sufficient time for IJs to recover and develop into mixed stages of non-IJs and adults. *Acrobeles* sp. (stain PS1156), *C. elegans* (strain N2), and *P. pacificus* (strain PS312) were received from the Caenorhabditis Genetics Center and cultured on NGM agar with *Escherichia coli* OP50 using standard methods (Brenner, 1974).

*Pratylenchus penetrans, A. avenae*, and *M. hapla* were extracted from Petri dishes using a Baermann funnel and washed three times with distilled water before fixation. Second stage juveniles (J2s) of *H. glycines* were extracted from soybean roots using sugar centrifugation and washed three times with distilled water (Jenkins, 1964). *C. elegans, Acrobeles* sp., and *P. pacificus* were washed from the Petri dishes and rinsed three times with M9 buffer (Brenner, 1974) to remove adhering bacteria before fixation. IJs of *H. bacteriophora, H. megidis, S. carpocapsae*, and *S. glaseri* were washed three times with distilled water before fixation.

### DAPI Staining

**FIGURE 2:**
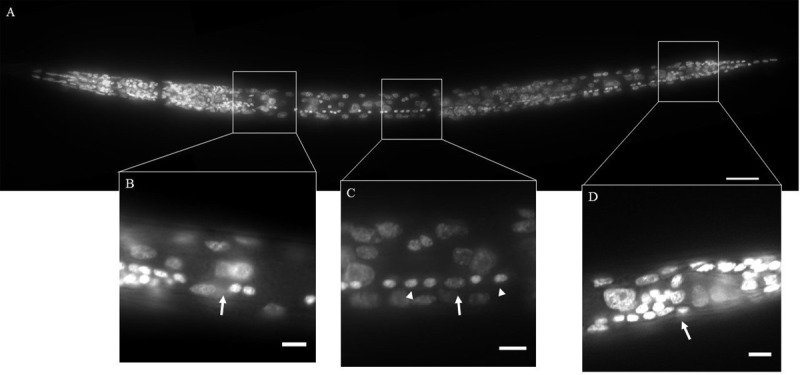
**DAPI staining of wild-type *Caenorhabditis elegans* (Clade 9).** The ventral nerve cord (VNC) consists of a line of motor neurons extending along the ventral midline from the retrovesicular ganglion (RVG) to the pre-anal ganglion (PAG). **(A)** Ventral view of DAPI stained animal. **(B)** Region surrounding end of RVG and anterior portion of VNC (arrow). **(C)** Part of the VNC showing neuronal (arrowheads) and hypodermal (arrow) nuclei. **(D)** Division between PAG and VNC (arrow). Inset scale bars = 5 μm.

Nematodes were fixed in 4% formaldehyde at 4°C overnight in microcentrifuge tubes. Following formaldehyde fixation, nematodes were washed three times with Phosphate buffered saline with Triton X-100 (PBST; 0.1% Triton) and incubated in methanol for at least 4 h. Nematodes were then washed three times with PBST and incubated in 0.2–0.5 μg/ ml of 4′, 6-diamidino-2-phenylindole (DAPI; Life technologies, Carlsbad, CA, USA) overnight in dark at room temperature. Nematodes were store at 4°C prior to examination. We were unable to distinguish the sex of *H. glycines, M. hapla* or young juveniles of *P. penetrans*. The gender of *Steinernema* was identified based on the shape of the gonad. Only hermaphrodites of *C. elegans* and *P. pacificus*, and females of *Acrobeles* sp. were examined. Between 10 and 30 animals were examined for each species. Putative neurons in the VNC were identified based on the size and morphology of the nuclei (Sulston, 1976; White et al., 1976). Counts of neuronal nuclei were made from immediately posterior of the retrovesicular ganglion (RVG) to immediately anterior of the preanal ganglion (PAG) (**Figure 2**). In cases where a cell could not be unambiguously identified as a neuron an independent count was made by a researcher blind to the species. If the cell identity was still in doubt, it was excluded from the total neuron count.

A separate microwave fixation method was developed for the staining of J2 *M. hapla.* Nematodes were recovered from tomato root cultures and transferred to 0.2X Finney-Ruvkun buffer with 5% methanol and 2% formaldehyde (http://www.wormatlas.org/EMmethods/Antibodystaining.htm; Finney and Ruvkun, 1990). The fixation solution was placed in a 1 L ice bath in a household microwave with rotating turntable. A separate 1 L beaker of H_2_O was included as a heat sink. Nematodes were exposed to three separate 1 min irradiations at 30% power with a 30°C maximum temperature. Following fixation, nematodes were washed three times with PBST and then irradiated nine times in 0.2 μg/ml DAPI for 3–4 min at 30% power with a maximum temperature of 39°C.

### Dye-Filling

Dye-filling was adapted from previously described methods (Tong and Bürglin, 2010). All nematodes were transferred into centrifuge tubes and prewashed 3 times with distilled water. Nematodes were incubated in 10 μg/ml of DiI (1,1′-Dioctadecyl-3,3,3′,3′-tetramethylindocarbocyanine perchlorate; Life technologies, Carlsbad, CA, USA) and wrapped with aluminum foil on an orbital shaker for at least 2 h. Excess liquid was removed from the centrifuge tubes and nematodes were transferred onto 1.5% water agar for at least 1 h covered with foil to remove excess dye. Animals were then picked to agar pads amended with 20 mM levamisole for imaging with DIC and fluorescent microscopy (Shaham, 2006). For each species, more than 30 animals were examined. Images were acquired using a Zeiss M2 AxioImager with mechanized stage and Zen software. Z-projections were created using FIJI.

### Development of the VNC in *A. avenae*

Synchronized *A. avenae* eggs were obtained by picking gravid *A. avenae* females into 5% M9 buffer (Stiernagle, 2006) for 1 h to lay eggs. Adults were then removed and the remaining eggs stored at 22°C until hatching (∼48 h). Immediately after hatching, J2s were transferred to 1/8 strength Potato Dextrose Agar with the fungus *Phomopsis logicolla* at 22°C. *A. avenae* nematodes were then examined at specified time points after hatching by mounting on a 5% agar pad with 20 mM levamisole and observed using DIC microscopy.

## Results

### Surprising Variation in the Number of Putative VNC-Neurons Among Nematode Species

The VNC is an easily recognized series of neurons lying along the ventral cord of nematodes (**Figure 2**). In *C. elegans*, the VNC contains a series of 57 motorneurons lying between the RVG and the PAG (Sulston, 1976; White et al., 1976). Using DIC optics, neuronal nuclei in *C. elegans* are typically small, granular in appearance and lacking obvious nucleoli (Sulston, 1976; Yochem, 2006). Neurons are seen as highly condensed round fluorescent puncta following DAPI staining (Sulston, 1976).

**FIGURE 3:**
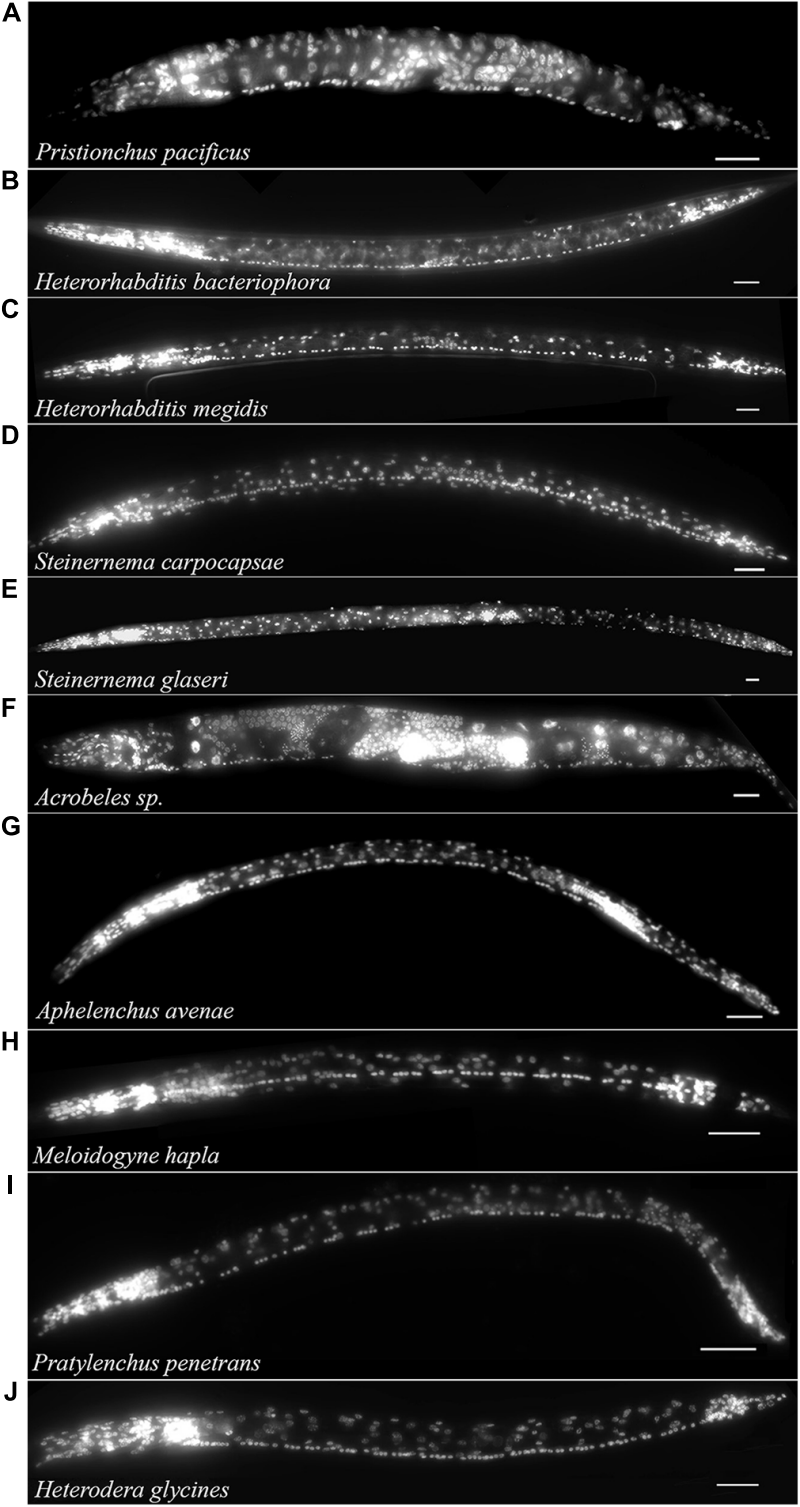
**The VNC of nematodes in Clades 8–12 is highly variable.** Fluorescent micrographs of individual nematode species fixed in formaldehyde and exposed to DAPI followed by imaging under fluorescent light. Species examined include: **(A)** *Pristionchus pacificus* hermaphrodite (Clade 9). **(B)** *Heterorhabditis bacteriophora* infective juvenile (Clade 9). **(C)** *Heterorhabditis megidis* infective juvenile (Clade 9). **(D)** *Steinernema carpocapsae* infective juvenile (Clade 10). **(E)** *Steinernema glaseri* infective juvenile (Clade 10). **(F)** *Acrobeles* sp. adult female (Clade 11). **(G)** *Aphelenchus avenae* J3 (Clade 12). **(H)** *Meloidogyne hapla* J2 (Clade 12). **(I)** *Pratylenchus penetrans* adult female (Clade 12). **(J)** *Heterodera glycines* J2 (Clade 12). Scale bar = 20 μm.

The free-living nematode, *P. pacificus* (Clade 9) is used as a satellite nematode species for evolutionary studies (Sommer, 2005, 2006). Similar to *C. elegans, P. pacificus* typically feeds on bacteria. However, *P. pacificus* is also capable of predatory behavior toward other nematodes (Bumbarger et al., 2013). We found that the *P. pacificus* ventral cord contained approximately 20% fewer VNC neurons than *C. elegans* (**Table 1**; **Figure 3A**) suggesting that the number of neurons does not strictly correlate with phylogeny. Furthermore, this data indicates that evolved behaviors such as predation do not necessarily require an increase in the number of motorneurons. To test if other, more distantly related bacterial feeding nematodes, show even greater divergence in ventral cord neuron number, we examined the Clade 11 bacterial feeding nematode *Acrobeles* sp. Interestingly, *Acrobeles* sp. showed a nearly identical number of VNC neurons as *C. elegans* (**Table 1**; **Figure 3F**).

**Table 1:** Ventral cord neuron cell bodies in nematode species.

Clade	Species	Number of neurons	Sample size	Range	Stage
8	*Ascaris suum*^1^	55	NA	NA	Adult
9	*Caenorhabditis elegans*^2^	57	NA	NA	Post J1
	*Heterorhabditis bacteriophora*	59	22	57–62	IJ
	*Heterorhabditis megidis*	59	17	54–61	IJ
	*Pristionchus pacificus*^3^	46	18	43–48	Post J2
10	*Steinernema carpocapsae*	76	15	72–79	IJ
	*Steinernema glaseri*	76	11	74–80	IJ
11	*Acrobeles* sp.	57	10	54–59	Mixed
12	*Aphelenchus avenae*^4^	66	10	63–69	Post J2
	*Meloidogyne hapla*	65	10	62–68	J2
	*Pratylenchus penetrans*	57	20	53–58	Mixed
	*Heterodera glycines*	66	14	62–69	J2

*IJ, infective juveniles and J2, second stage juvenile stage. ^1^Species not examined in this study, number of neurons obtained through light microcopy (Stretton et al., 1978). ^2^Species not examined in this study, numbers of neurons obtained through electron and light microscopy (Sulston, 1976; White et al., 1976). ^3^Young J2s of *P. pacificus* have 20 neurons in the VNC. ^4^Young J2s of *A. avenae* have 26 neurons in the VNC (11 nematodes observed using Differential Interference Contrast).*

The entomopathogenic nematode genera *Heterorhabditis* sp. (Clade 9) and *Steinernema* sp. (Clade 10) infect a wide range of insect hosts. Though phylogenetically distinct, the two genera have similar lifestyles. *Heterorhabditis* sp. is more closely related to *C. elegans* than to *Steinernema* sp. (Clade 10). We found that two species of *Heterorhabditis* had 59 ventral cord neurons, similar to *C. elegans* (**Table 1**; **Figure 3B**). However, the ventral cord of *S. carpocapsae* contained 76 neurons (**Table 1**; **Figure 3D**); approximately 40% more than *C. elegans* and the largest number of VNC neurons among all examined species. *S. carpocapsae* is capable of an unusual jumping behavior wherein it stands on its tail, curls to form a loop and quickly extends to jump into the air (Reed and Wallace, 1965). We hypothesized that the increased number of neurons in *S. carpocapsae* evolved to allow for jumping behavior. To test this, we examined *S. glaseri*, another entomopathogenic species that does not exhibit jumping behavior. While slightly longer than *S. carpocapsae, S. glaseri* also had 76 neurons in the VNC (**Table 1**; **Figure 3E**). This data suggests either that jumping behavior does not specifically require additional motor neurons or that an ancestor to *S. glaseri* could jump and the additional neurons in *S. glaseri* are remnants of this ancestor. *Steinernema* sp. are male-female species. During the IJ stage sexes are easily distinguishable based on the shape of the gonad. However, we did not observe major differences in the number of VNC nuclei between sexes (data not shown).

Clade 12 contains nematodes with diverse life histories including fungal-feeding, plant-parasitic, and insect-parasitic species (van Megen et al., 2009). We examined four species in Clade 12 including one fungal-feeding species and three plant-parasitic species. There was no obvious correlation between the number of VNC neurons and food source or phylogeny. The fungal-feeding nematode *Aphelenchus avenae* and the plant-parasitic nematodes, *Heterodera glycines* and *Meloidogyne hapla* each had approximately 65 VNC neurons (**Table 1**; **Figures 3G,H,J**). However, the plant-parasitic nematode *P. penetrans* ventral cord contained fewer neurons than any other Clade 12 species (**Table 1**; **Figure 3I**). *Pratylenchus* is considered basal to *Meloidogyne* (van Megen et al., 2009). This data suggests either that there were multiple events leading to an increase in VNC number or that *Pratylenchus* underwent a loss in VNC neurons during evolution.

### Neuronal Heterochrony has Evolved At Least Twice Among Nematodes

*Caenorhabditis elegans* does not hatch with a full set of VNC neurons. Following embryogenesis, *C. elegans* hatches as a J1 (equivalent to L1 in *C. elegans* nomenclature) with 15 VNC neurons (Sulston, 1976). During J1 development, additional precursor cells (P0–P12 cells) migrate into the ventral cord followed by multiple rounds of cell division to produce the final complement of 57 VNC neurons (Sulston, 1976). This post-embryonic development occurs entirely within the J1–J2 developmental period. To determine if post-embryonic VNC development during J1 development is conserved, we examined the VNC of three species that hatch as J2s rather than J1s. We examined the VNC of both newly hatched J2s and adult nematodes of *P. pacificus* (Clade 9), *A. avenae* (Clade 12) and *P. penetrans* (Clade 12). Both *P. pacificus* and *A. avenae* showed delayed development in the VNC. *P. pacificus* hatched as a J2 with 20 VNC neurons while the adult hermaphrodite has 45 VNC neurons (**Table 1**). Similarly, *A. avenae* hatched as a J2 with 26 VNC neurons, while the adult female has 66 (**Table 1**). Interestingly, the delayed development of the VNC was not conserved among all species that hatch as J2s. We found no apparent difference in the number of VNC neurons between newly hatched J2 and adult *P. penetrans* (Clade 12) (data not shown). These data suggest an independent evolution of neuronal heterochrony in nematodes.

**FIGURE 4:**
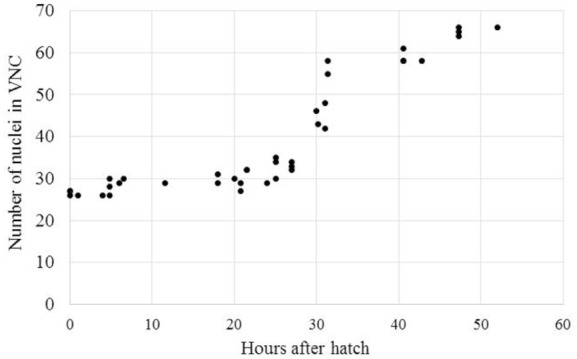
***Aphelenchus avenae* undergoes a post-hatch increase in the number of VNC neurons during J2.** The number of VNC nuclei was examined with DIC microscopy in synchronized *A. avenae* nematodes at various time-points following hatch. The molt from J2 to J3 occurs at approximately 36 h after hatching. Each data point represents an individual animal.

The delayed development seen in *A. avenae* and *P. pacificus* may be due to a shift in development from the *C. elegans-*like J1 VNC development to a J2 VNC development. Alternatively, the delayed development may be due to a progressive increase in VNC neuron number from J1 to the adult stage. To test these options, we collected time-series data on the development of the VNC in *A. avenae* using DIC microscopy. *Aphelenchus avenae* is an easily cultured fungal feeding nematode, closely related to, and a possible transitional model for, plant-parasitic nematodes. We found that eggs developed from single-cell embryos to hatched J2s in approximately 48 h. We observed 26 neurons in the VNC of newly hatched J2s using DIC microscopy. Cell division occurs after hatching and during feeding. Immediately prior to the J3 molt (25–35 h after hatching), we observed the most intensive increase of neurons in the VNC. By 48 h after hatching, *A. avenae* had a fully developed VNC with 66 neurons (**Table 1**; **Figures 3** and **4**). Thus, the delayed development of the VNC in *A. avenae* is a shift from a *C. elegans-*like J1 developmental sequence to a J2 post-hatching developmental sequence.

### Sensory Neuron Dye-Filling Varies Among and Within Nematode Clades and Among Developmental Stages

**FIGURE 5:**
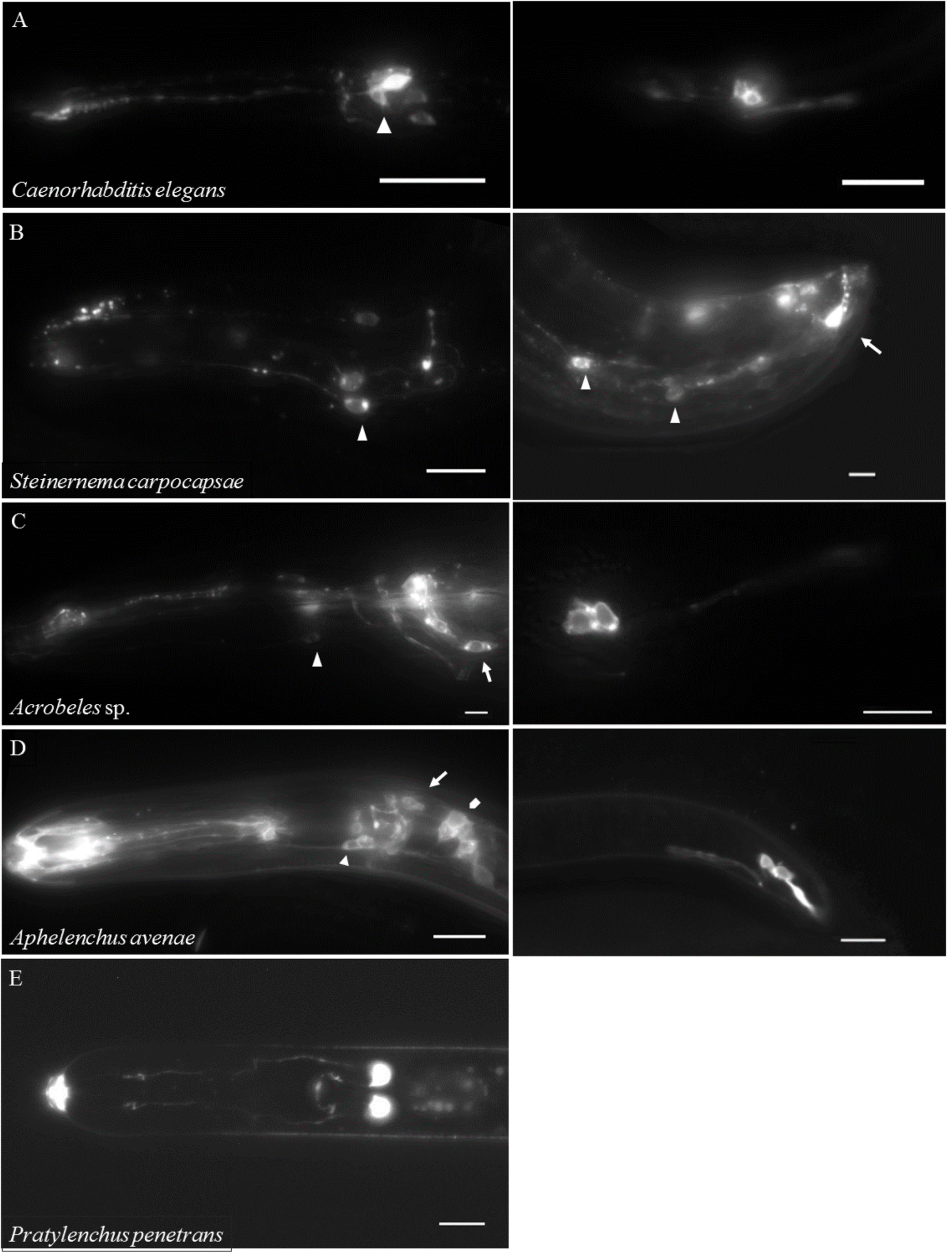
**DiI-filling is highly variable among nematodes.** Live nematodes were exposed to DiI for 2 h followed by repeated washes in water or buffer and then imaged with fluorescent microscopy. **(A)** Left Anterior of the Clade 9 nematode *Caenorhabditis elegans* dauer, arrowhead indicates amphid neurons. **(A)** Right Posterior of a *Caenorhabditis elegans* dauer, two pairs of phasmid neurons are shown. **(B)** Left Anterior of the Clade 10 nematode *Steinernema carpocapsae* female, arrow indicates inner labial neurons. **(B)** Right Posterior of a *S. carpocapsae* male, arrow indicates phasmid neurons and arrowheads indicate unidentified neurons. **(C)** Left Anterior of the Clade 11 nematode *Acrobeles* sp. female, arrow indicates amphid neurons and arrowhead indicates inner labial neurons. **(C)** Right Posterior of an *Acrobeles* sp. female, two pairs of phasmid neurons are shown. **(D)** Left Anterior of the Clade 12 nematode *Aphelenchus avenae*, pentagon indicates amphid neurons, arrow indicates cephalic neurons, and arrow head indicates inner labial neurons. **(D)** Right Posterior of an *Aphelenchus avenae* female, two pairs of phasmid neurons are shown. **(E)** Anterior of the Clade 12 nematode *Pratylenchus penetrans* female (ventral view), one pair of amphid neurons in the anterior of the nematode is shown. Scale bar = 10 μm for all images.

We were interested if a similar divergence in neuronal properties could be detected using a dye-filling protocol common to *C. elegans* research. Specific ciliated sensory neurons in the head and tail of *C. elegans* will fluoresce following exposure to the lipophilic compound DiI (Hedgecock et al., 1985; Collet et al., 1998). In *C. elegans*, six pairs of amphid neurons in the head and two pairs of phasmid neurons in the tail can be stained using fluorescent dyes (DiI, DiO and FITC; **Figure 5A**; Hedgecock et al., 1985; Collet et al., 1998). Staining patterns can vary depending on the method. For example, under certain conditions six inner-labial sensory neurons can also be stained (Tong and Bürglin, 2010). While the precise mechanism is unknown, dye-filling is frequently used to indicate the structural integrity of specific sensory neurons in *C. elegans* (Perkins et al., 1986). For example, Srinivasan et al. (2008) used a comparative dye-filling approach to identify and investigate the function of a homologous sensory neuron among six free-living nematodes in Clades 9 and 10. They found a nearly identical dye-filling pattern among all tested nematodes, suggesting a high degree of conservation of nematode neuroanatomy. We expanded upon these results by testing additional species in Clades 9–12.

We initially attempted dye-filling on the entomopathogenic nematodes *Heterorhabditis bacteriophora* (Clade 9) and *Steinernema carpocapsae* (Clade 10). These entomopathogenic nematodes infect their hosts as IJs, a non-feeding developmentally arrested stage analogous to the *C. elegans* dauer stage. Following infection, the nematodes will resume development and feed until resources are depleted. The nematodes then reenter the IJ stage and disperse to find a new host. Interestingly, we did not observe any dye-filling in IJs of either species. Therefore, we isolated non-infective stages of these species using both *in vitro* and *in vivo* methods. We observed dye-filling in six putative IL2 orthologs in non-IJs and adults of *S. carpocapsae* (**Figure 5B**). Two pairs of putative phasmid neurons also dye-filled in the tail of males of *S. carpocapsae* (**Figure 5B**). Surprisingly, dye-filling of amphid neurons was only periodically observed in both of these insect-parasitic species. Based on these results, we examined the dauer stage of *C. elegans.* As previously shown, five of the six amphid neurons routinely dye-filled in *C. elegans* dauers (**Figure 5A**) (Peckol et al., 2001). However, we never observed IL2 dye-filling in *C. elegans* dauers using modified dye-filling protocols that routinely result in IL2 dye-filling in non-dauers. This lack of dye-filling may be due to modifications to cilia structure during dauer as previously shown by TEM (Albert and Riddle, 1983). Together these results demonstrate that development has a marked influence on sensory neuron properties.

Similar to our VNC data, we found significant variability in dye-filling among nematode clades. As discussed above, *H. bacteriophora* (Clade 9) and *S. carpocapsae* (Clade 10) showed different dye-filling patterns than *C. elegans.* However, the bacterial-feeding nematode *Acrobeles* sp. (Clade 11) showed an identical dye-filling pattern to *C. elegans* (**Figure 5C**). The fungal-feeding nematode *A. avenae* (Clade 12) also displayed a similar dye-filling pattern as *C. elegans*. Specifically, six putative IL2 orthologs and twelve putative amphid orthologs dye-filled in *A. avenae* (**Figure 5D**). In *A. avenae*, an additional four neurons were stained anterior to amphid neurons that we identified as cephalic neurons. Previous TEM data demonstrated that *A. avenae* females have two cephalic neurons (CEP1 and CEP2) in each sensillum (Ragsdale et al., 2009). The cilia of CEP1 in *A. avenae* are exposed to the environment, whereas CEP2 is embedded in the cuticle. Therefore, the dye-filling of cephalic neurons in *A. avenae* corresponds to the EM data. In *C. elegans* hermaphrodites, there is one cephalic neuron in each sensillum that is not exposed to the external environment and does not dye-fill (Hedgecock et al., 1985). However, *C. elegans* males, similar to *A. avenae* females, have two cephalic neurons. While these male-specific cephalic neurons (CEM) in *C. elegans* are exposed to the external environment, they do not dye-fill (Perkins et al., 1986).

Unlike the fungal feeding Clade 12 nematode *A. avenae*, the Clade 12 plant-parasitic nematodes displayed restricted dye-filling. In *P. penetrans*, only one pair of putative amphid neurons stained (**Figure 5E**). Based on axon morphology and nuclear position we identified these neurons as likely ADL orthologs. We observed only occasional and weak dye-filling in *P. penetrans* phasmid neurons (data not shown). Two plant-parasitic nematodes, *H. glycines* and *M. hapla*, did not show dye-filling in any neuron suggesting a possible modification of these neurons.

## Discussion

Nematodes have made substantial contributions to our understanding of the molecular basis of evolution. These studies have generally focused on non-neuronal structures such as vulva development and male-tail morphology (Fitch, 1997; Sommer, 2000). Fewer studies have utilized nematodes for examining the evolution of neurodevelopment and neuroanatomy. The lack of nematode “evo-neuro” studies may be due, in part, to the assumption that the neuroanatomy of nematodes is highly conserved. The few comparative studies have primarily utilized low-throughput TEM to dissect ultrastructural differences in the sensory or pharyngeal nervous systems of a handful of species. Here, we demonstrated that light microscopy can be used to observe differences in neuroanatomy and neurodevelopment across multiple species. Furthermore, we showed that neuroanatomical and neurodevelopmental differences among nematodes are more abundant than previously assumed.

We found differences in the number and developmental timing of the VNC among various nematode species. The VNC of *C. elegans* and *A. suum* consist of a series of motor neurons that regulate movement through coordinated excitation and inhibition of body wall muscles. Amazingly, these phylogenetically and ecologically separate species show a nearly identical number and pattern of cholinergic and GABAergic VNC neurons (Stretton, 1976; White et al., 1976; Stretton et al., 1978; Johnson and Stretton, 1985, 1987; Mclntire et al., 1993; Duerr et al., 2001). As we found that several species diverge from the *C. elegans* and *Ascaris* neuroanatomy, it will be useful to examine the neurotransmitter identity of VNC neurons in these species for conservation of the basic patterning.

While identification of neuronal nuclei in the VNC is relatively straightforward using DAPI fluorescence and DIC microscopy there are two potential causes of ambiguity and resulting variation. In some species, a clear demarcation did not exist between the VNC and the retrovesicular and PAGs. This may have led to some of the variation in the number of VNC neurons recorded among individuals within a species. A similar variability in the position of individual VNC neurons is seen in *C. elegans* (White et al., 1976). An additional source of variability within species may be due to misidentification of cell type. While cell types were usually unambiguous, occasionally nuclei could not be strictly categorized as neuronal or non-neuronal. This ambiguity occurred most frequently in earlier larval stages where nuclei were often immediately adjacent to one another. In these cases, we used a second observer, blind to the species, to categorize the nucleus. In cases where the nucleus type was ambiguous to both researchers, the nucleus in question was excluded from the count. Therefore, it is possible that some of our counts were underestimates of the true number of VNC neurons. It will be interesting to confirm these counts and conduct comparative connectomics on select species with TEM.

The variation in nematode neuroanatomy did not strictly correlate with phylogeny. While more closely related to *C. elegans* (Clade 9) than *A. suum* (Clade 8), *P. pacificus* (Clade 9) had 20% fewer VNC neurons than either species. Similarly, *P. penetrans* had 20% fewer VNC neurons than all other examined Clade 12 species (van Megen et al., 2009). The number of VNC neurons does appear to be conserved within individual genera as shown by *Steinernema* sp. and *Heterorhabditis* sp. Similarly, sensory neuron dye-filling did not correlate with phylogeny. While *C. elegans* (Clade 9) and *Acrobeles* sp. (Clade 11) show a nearly identical dye-filling pattern, the entomopathogenic nematode *Heterorhabditis bacteriophora* (Clade 9) showed striking differences. This variability in dye-filling is consistent with previous results showing only a single pair of amphid neurons dye-fill in the Clade 10 mammalian-parasitic nematode *Parastrongyloides trichosuri* (Zhu et al., 2011). Although the precise mechanism of dye-filling is unknown, we hypothesize that differential dye-filling patterns indicate ultrastructural or biochemical differences among sensory neurons. Alternatively, the sensilla pores may be blocked or filled with secretions in certain species (Perry, 1996). However, we think this alternative hypothesis less likely. Previous TEM studies on *Heterodera glycines* and *Meloidogyne* sp. clearly indicate that the amphids are exposed to the environment (Wergin and Endo, 1976; Endo, 1980). *Heterodera glycines* (Clade 12), which showed no dye-filling with DiI, was previously shown to undergo amphid dye-filling with fluorescein isothiocyanate (Winter et al., 2002). In *C. elegans*, certain environmentally exposed amphid neurons do not dye-fill (Hedgecock et al., 1985). Again, this implies that ultrastructural or biochemical differences in individual neurons may underlie species–specific dye-filling in our study. Finally, we demonstrate that there are developmental differences in dye-filling within individual species. In *C. elegans* dauers, changes in the ultrastructure of individual neurons likely result in altered dye-filling during this developmental stage (Albert and Riddle, 1983; Peckol et al., 2001; Schroeder et al., 2013).

In *C. elegans*, post-embryonic development of the VNC requires the migration and subsequent cell division of 12 sub-ventral precursor cells (P cells). Following migration, the P cells divide to form an anterior neuroblast cell (Pn.a) and a posterior cell (Pn.p) which either forms a hypodermal nucleus or a vulval precursor cell. Certain ancestors of the *C. elegans* Pn.a cell undergo programmed cell death eventually leading to the defined 57 VNC neurons. Previous lineage analysis in *P. pacificus* demonstrated that the non-vulva forming Pn.P cells undergo apoptosis prior to hatch (Sommer and Sternberg, 1996). As *P. pacificus* and *C. elegans* have an identical number of Pn.a neuroblast cells (Félix et al., 2000), it seems likely that additional apoptotic events occur in the *P. pristionchus* Pn.a lineage following migration. Similarly, with species showing additional neurons, it will be important to find if this results from the embryonic development of additional P cells or through extra rounds of cell division.

Heterochrony is defined as differences in the timing of developmental events and may play an important role in evolution (Gould, 1977; Alberch et al., 1979; Smith, 2003). The delayed development of neurons in the VNC of *A. avenae* (Clade 12) and *P. pacificus* (Clade 9) is suggestive of neuronal heterochrony. As *P. penetrans* (Clade 12), which also hatches as a J2, shows no post-hatch VNC development we propose that the heterochronic developmental events in *A. avenae* and *P. pacificus* arose independently. In *C. elegans*, several mutants have been isolated that result in altered timing of development events (Ambros and Horvitz, 1984; Ambros, 1988; Lee et al., 1993). The homologs of these heterochronic genes and microRNAs in *C. elegans* are present throughout the animal kingdom and may play roles in altered developmental timing (Moss and Tang, 2003).

Our data suggest that the neuroanatomy of nematodes is not as highly conserved as previously described. The evolution of nematode nervous systems has been relatively neglected, in part, due to previous assumptions of high anatomical conservation. It will be valuable to use higher resolution imaging techniques and antibody staining to define the neuronal subtypes found in these species. For example, Stretton et al. (1978) found that the VNC of *A. suum* consists of five repeating sets of neurons each containing 11 cells. Among these cells, they found seven neuron types based on synaptic connectivity (Stretton et al., 1978). In our study, we observed a possible repeating pattern in the VNC of *M. hapla* (**Figure 3H**). However, our methods do not allow for the identification of individual neuron types. TEM could be used to study the neuron types in order to classify repeated segments on select species. In addition, immunohistochemistry may be utilized to distinguish the neurotransmitter identity of individual cells. Using immunohistochemistry, Loer and Rivard (2007) found variation in the expression of serotonin among the VNC of male Rhabditid nematodes. It will be interesting to examine the VNC for immunoreactivity to neurotransmitters such as serotonin and GABA. Together with previous data demonstrating large-scale rewiring between the pharyngeal nervous systems of *C. elegans* and *P. pacificus* (Bumbarger et al., 2013), we propose that the phylum Nematoda represent a bountiful source of data for understanding the evolution of nervous systems. A close comparative examination of the nervous systems of parasitic nematodes may also lead to the development of species–specific control strategies.

## Author Contributions

ZH and NS conceived project and designed experiments. ZH, SB, and NS performed experiments and analyzed results. ZH, SB, and NS wrote the paper.

## Conflict of Interest Statement

The authors declare that the research was conducted in the absence of any commercial or financial relationships that could be construed as a potential conflict of interest.
