# Corrigendum: Unexpected Variation in Neuroanatomy among Diverse Nematode Species

**DOI:** 10.3389/fnana.2016.00052

**Published:** 2016-05-03

**Authors:** Ziduan Han, Stephanie Boas, Nathan E. Schroeder

**Affiliations:** ^1^Department of Crop Sciences, University of Illinois at Urbana-ChampaignUrbana, IL, USA; ^2^Neuroscience Program, University of Illinois at Urbana-ChampaignUrbana, IL, USA

**Keywords:** invertebrate, amphid, phasmid, *Pratylenchus*, *Meloidogyne*, *Heterodera*, *Heterorhabditis*, heterochrony

The complete number of ventral nerve cord (VNC) neurons for *Ascaris suum* should be given as 72 in the introduction and Table 1. The 55 VNC neurons listed in our publication refers only to the five repeating segments of 11 neurons as discussed in Stretton et al. (1978). An additional 17 neurons are found outside of these repeating segments within the *A. suum* VNC (see Stretton and Maule, 2013 for review).

This correction does not impact the results or the conclusions reached in our study.

## Author contributions

All authors listed, have made substantial, direct and intellectual contribution to the work, and approved it for publication.

### Conflict of interest statement

The authors declare that the research was conducted in the absence of any commercial or financial relationships that could be construed as a potential conflict of interest.
